# Modulating Catalytic Activity and Durability of PtFe Alloy Catalysts for Oxygen Reduction Reaction Through Controlled Carbon Shell Formation

**DOI:** 10.3390/nano9101491

**Published:** 2019-10-19

**Authors:** Youngjin Kim, A. Anto Jeffery, Jiho Min, Namgee Jung

**Affiliations:** Graduate School of Energy Science and Technology (GEST), Chungnam National University, 99 Daehak-ro, Yuseong-gu, Daejeon 34134, Korea; yoongjin123@naver.com (Y.K.); jeffeeanto@gmail.com (A.A.J.); mjh9780@naver.com (J.M.)

**Keywords:** PtFe alloy, carbon shell, annealing, oxygen reduction reaction, durability, fuel cell

## Abstract

Demand on synthetic approaches to high performance electrocatalyst with enhanced durability is increasing for fuel cell applications. In this work, we present a facile synthesis of carbon shell-coated PtFe nanoparticles by using acetylacetonates in metal precursors as carbon sources without an additional polymer coating process for the carbon shell formation. The carbon shell structure is systematically controlled by changing the annealing conditions such as the temperature and gas atmosphere. PtFe catalysts annealed at 700 °C under H_2_-mixed N_2_ gas show much higher oxygen reduction reaction (ORR) activity and superior durability compared to a Pt catalyst due to the ultrathin and porous carbon shells. In addition, when increasing the annealing temperature, the carbon shells encapsulating the PtFe nanoparticles improves the durability of the catalysts due to the enhanced crystallinity of the carbon shells. Therefore, it is demonstrated that the developed hybrid catalyst structure with the carbon shells not only allows the access of reactant molecules to the active sites for oxygen reduction reaction but also prevents the agglomeration of metal nanoparticles on carbon supports, even under harsh operating conditions. The proposed synthetic approach and catalyst structure are expected to provide more insights into the development of highly active and durable catalysts for practical fuel cell applications.

## 1. Introduction

Polymer electrolyte membrane fuel cells (PEMFCs) have been regarded as promising power sources owing to their interesting features such as high power density and energy conversion efficiency, as well as an eco-friendly reaction mechanism [1,2]. However, in PEMFCs, since the oxygen reduction reaction (ORR) at the cathode is much slower than the hydrogen oxidation reaction (HOR) at the anode, tremendous research efforts have been focused on developing highly-efficient and active catalysts for the ORR [3]. The state-of-the art catalyst used so far in PEMFCs is Pt, however, it still has many problems in practical fuel cell applications due to its high cost, scarcity, and poor long-term stability. The poor durability of Pt catalyst originates from harsh fuel cell operation conditions in an acidic medium leading to agglomeration, detachment, and particle dissolution in high operating voltage range, thus limiting its wide commercialization [4,5,6]. Besides the durability issues, the catalytic activity needs to be greatly enhanced since the use of expensive Pt needs to be reduced. Therefore, various approaches have been extensively developed, which include alloying Pt with 3*d* transition metals (M = Fe, Co, and Ni) [7,8,9,10,11,12,13,14,15], core-shell nanostructures [16], and modification of support materials [17,18]. 

Among them, alloying of Pt with transition metals to form a bimetallic structure, Pt−M, has advantages of showing greatly improved activity for the ORR due to the modified electronic structure and physico-chemical properties [3]. On the other hand, these alloy catalysts show much less durability compared to Pt since their 3*d* transition metals are prone to dissolution in acidic electrolytes when they are exposed to high potential during long-term operation [19,20,21]. To circumvent this problem, there are several studies devoted to improving the durability of Pt−M alloy catalysts [22,23]. One such interesting strategy is to form carbon shells on Pt−M alloy surfaces as a barrier layer to prevent the metal dissolution [24]. There are several reports that describe the carbon shell formation on Pt−M alloy nanoparticles. However, the synthetic approaches necessarily involve an additional polymer coating procedure to provide carbon sources for the carbon shell formation and a high-temperature carbonization step [25]. In addition, it is very difficult to reliably control the thickness and porosity of the carbon shell on Pt−M because the physical structure of the carbon shell might be strongly affected by the chemical property of the polymer used and the coating and carbonization conditions. 

In this work, we developed a novel strategy to form a well-defined carbon shell on Pt−M alloy nanoparticles simply by using acetylacetonates in Pt(acac)_2_ and Fe(acac)_3_ precursors as carbon sources without additional polymer coating process. A simple heat treatment after a solution-based thermal decomposition of the metal precursors resulted in the fabrication of thin carbon shell-coated PtFe nanoparticles. Furthermore, the structure of carbon shells was systematically controlled by changing the annealing conditions such as the temperature and gas atmosphere. The electrochemical properties of the developed catalysts were intensively studied to evaluate the effect of carbon shell formation on their ORR activity and durability. We believe that this strategy is an effective way to construct the controlled formation of carbon shells on PtFe catalysts with promising advantages of enhanced activity and durability in harsh fuel cell conditions, and also provides insights into the design of novel structures of metal-carbon hybrid catalysts.

## 2. Materials and Methods 

### 2.1. Materials

All chemical reagents used were of analytical grade and used without further purification. Carbon blacks (Vulcan XC72, Cabot) were purchased from Cabot Inc., Alpharetta, GA, USA. Commercial Pt/C catalyst (Johnson Matthey) were used as a standard. 1-Octadecene (90%), platinum acetylacetonate (Pt(acac)_2_, 97%), iron acetylacetoante (Fe(acac)_3_, 97%), oleylamine (70%), nafion ionomoer (5 wt%), and 2-propanol (99.5%) were procured from Sigma-Aldrich Inc., St. Louis, MO, USA. *n*-Hexane (95%) and ethanol (95%) were acquired from Samchun Pure Chemical, Daejeon, Korea.

### 2.2. Synthesis of Carbon Shell-Coated PtFe Catalysts

For the synthesis of carbon shell-coated PtFe catalysts, 0.1 g of carbon black was well dispersed through ultrasonication in 15 mL of oleylamine and 160 mL of 1-octadecene for 20 min. Meanwhile, 0.044 g of Pt(acac)_2_, 0.24 g of Fe(acac)_3_, 5 mL of oleylamine, and 20 mL of 1-octadecene were taken in a separate vial and allowed to disperse until a highly homogeneous solution was formed. Afterwards, the two solutions were mixed well and further sonicated for 1–2 min, and then subjected to heating at 300 °C for 2 h under Ar atmosphere. After thermal decomposition reaction of Pt and Fe precursors, the solution temperature was cooled down to 80 °C. The obtained slurry containing catalyst was filtered and then washed with a copious amount of *n*-hexane and ethanol solution. The obtained product was dried in a vacuum oven at 60 °C overnight. The as-prepared catalyst was termed as PtFe_ASP. Afterwards, the as-prepared PtFe_ASP catalyst was further annealed for 1h at two different tempertures (700 and 900 °C) in Ar gas or 5% H_2_-mixed N_2_ gas, respectively, and they were formulated as PtFe 700_Ar, PtFe 700_H_2_, PtFe 900_Ar, and PtFe 900_H_2_, respectively. 

### 2.3. Electrochemical Measurements

All electrochemical measurements were tested in a standard three-electrode system using a rotating disk electrode (RDE, Metrohm, Switzerland) with a glassy carbon (GC, Metrohm, Switzerland) in Ar saturated 0.1 M HClO_4_ electrolyte. Electrocatalyt deposited GC, Pt wire, and Ag/AgCl electrode served as the working, counter, and reference electrodes, respectively. All potential values were represented versus the reversible hydrogen electrode (RHE). The catalyst ink was prepared by dispersing a mixture containing 5 mg of the prepared catalyst, nafion ionomoer (68.7 µL), and 2-propanol (500 µL) through ultrasonication for few minutes. A drop of catalyst ink (total metal loading = 44.86 µg cm^−2^) was applied to a GC electrode (geometric area = 0.196 cm^2^) and then dried at room temperature. Cyclic voltammograms (CVs) were measured by cycling the potential between 0.05 and 1.05 V_RHE_ at a scan rate of 20 mV s^−1^ in an Ar-saturated 0.1 M HClO_4_ solution. For ORR tests, the potential was applied between 0.05 and 1.05 V_RHE_ at a scan rate of 5 mV s^−1^ under a constant rotation speed of 1600 rpm in O_2_-saturated 0.1 M HClO_4_ solution. For CO stripping measurements, the working electrode potential was held at 0.05 V_RHE_ for 15 min while bubbling pure CO gas into 0.1 M HClO_4_. After purging the electrolyte with Ar gas for 20 min, the residual carbon monoxide molecules in the electrolyte were completely removed, and then, in Ar-saturated electrolyte, CV curves were obtained at a scan rate of 20 mV s^−1^ within potential range of 0.05 and 1.05 V_RHE_. The electrochemcial surface area (ECSA) was calculated by integrating the current in the CO oxidation peak region, assuming a monolayer CO charge of 420 µC cm^−2^. Accelerated durability test (ADT) was conducted in the potential range of 0.6 and 1.1 V_RHE_ in an O_2_ saturated 0.1 M HClO_4_ solution for 5000 cycles at scan rate of 100 mV s^−1^. CV, and the ORR and CO stripping were then measured again.

### 2.4. Physical Characterization

To estimate the metal loading of catalysts, thermogravimetric analysis (SII EXSTAR6000 TG/DTA6200, Ibaraki, Japan) was conducted. As a result, it was confirmed that all the catalysts similarly had 18–20 wt% metal loadings on carbon supports (Figure S1). Microstructure and morphologies of the commercial Pt/C and heat-treated PtFe catalysts were observed under a high-resolution transmission electronmicroscopy (HRTEM) (Tecnai G^2^ F30 S-Twin, FEI, Thermo Fisher Scientific, Eindhoven, Netherlands). The crystal structures and phase identification were analyzed using an X-ray diffractometer (XRD) (D/MAX-2200 Ultima, Rigaku International Corporation, Tokyo, Japan). The atomic composition of the PtFe catalyst was confirmed by energy-dispersive X-ray spectroscopy (EDX, Tecnai G^2^ F30 S-Twin, FEI Thermo Fisher Scientific, Eindhoven, Netherlands) analysis using the TEM. Surface structure and chemical composition were analyzed using an X-ray photoelectron spectrometer (XPS), Multilab 2000 spectrometer (Thermo Electron Corporation, Grinstead, UK), and Al Kα radiation (λ = 1486.6 eV).

## 3. Results and Discussion

**Figure 1:**
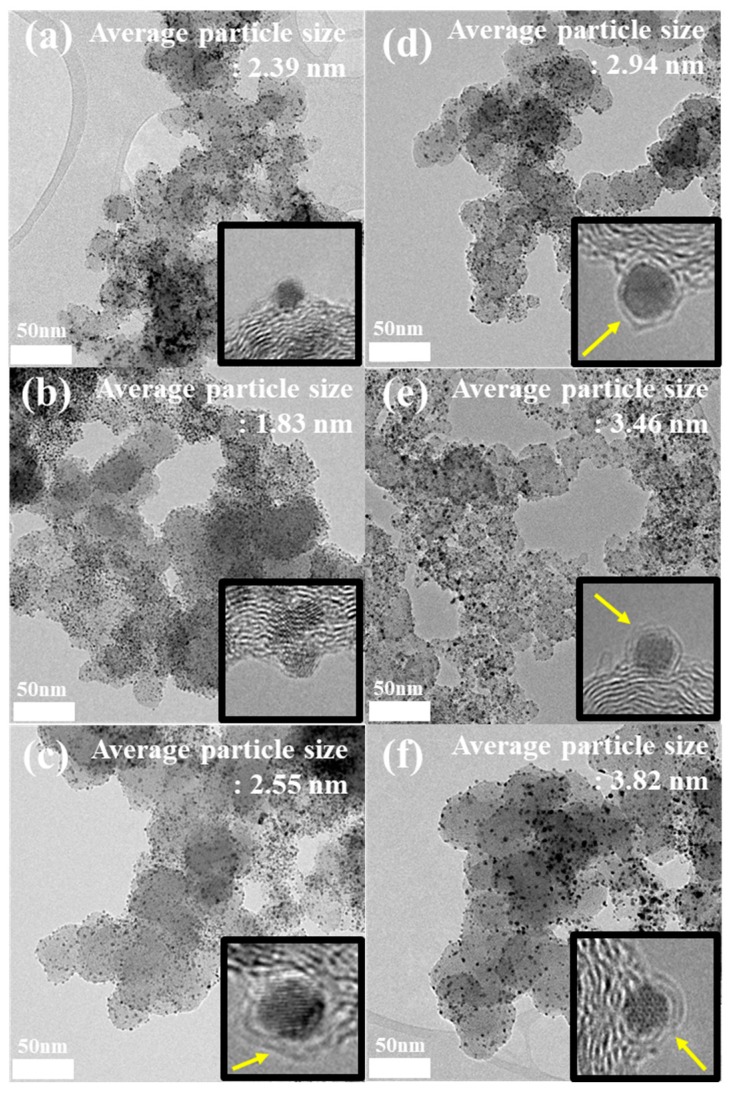
Transmission electronmicroscopy (TEM) images of (**a**) commercial Pt/C, (**b**) PtFe_ASP, (**c**) PtFe 700_Ar, (**d**) PtFe 700_H_2_, (**e**) PtFe 900_Ar, and (**f**) PtFe 900_H_2_. The insets show high-resolution transmission electronmicroscopy (HRTEM) images of the metal nanoparticles in the samples. Yellow arrows in the insets indicate the carbon shells coated on the PtFe nanoparticles.

As shown in Figure 1, the TEM images of Pt/C, PtFe_ASP, and PtFe heat-treated catalysts show a homogeneous distribution of PtFe nanoparticles on carbon support. The as-prepared PtFe_ASP catalyst (Figure 1b) exhibits clean metal surfaces similar with commercial Pt/C (Figure 1a) indicating that the carbon atoms dissolved inside PtFe lattice due to the carbon solubility in Pt and Fe making PtFe to appear as clean surfaces free from carbon coating [26,27]. In contrast to their surface structures, upon annealing at high temperatures, the dissolved carbon atoms segregated to the PtFe surface, resulting in well-defined carbon shell formation (yellow arrows in the insets of Figure 1c–f). A number of carbon shell-coated PtFe nanoparticles are identified in Figure S2. Since the high-temperature heat treatment at 700–900 °C induces the graphitization of the formed carbon shells [26], in the present case, we can assume that the heat-treated PtFe catalysts have graphitized carbon shell layers. Therefore, from the TEM analyses, it can be concluded that the formation of carbon shells on PtFe nanoparticles occurs through dissolution of carbon atoms into PtFe nanoparticles, followed by segregation of carbons to the surface and finally the formation of graphitized carbon shell layers during the high temperature annealing process [28,29,30]. The existence of the carbon shells on the nanoparticles can be also elucidated by the effects of high-temperature annealing process on the particle dispersion and size. When metal nanoparticles are heated to high temperature of 700–900 °C, they generally tend to agglomerate severely, resulting in the formation of much larger particles on the carbon supports. On the other hand, if the nanoparticles are encapsulated with rigid carbon shells, the particle agglomeration can be effectively prevented during the annealing process [24,25]. As shown in Figure S3, the negligible changes in the particle size of prepared catalysts even after the heat treatment indicate that the carbon shells were formed during the annealing process.

However, it is difficult to find distinguishable difference among the carbon shell-encapsulated PtFe nanoparticles fabricated under different annealing conditions due to the limited resolution of the TEM. Nevertheless, it is expected that the pore size of the carbon shells might be reduced due to their enhanced crystallinity after increasing the annealing temperature [31,32,33,34,35,36,37]. In addition, when the samples are heated at H_2_-mixed N_2_ gas atmosphere, the thickness of the carbon shells can decrease since H_2_ gas etches the activated carbon sites and decreases the growth rate of carbon layers during the formation of the graphitic carbon layer [38,39]. Accordingly, for PtFe 700_Ar and PtFe 900_Ar samples, thicker carbon shell layers might be obtained compared to those of PtFe 700_H_2_ and PtFe 900_H_2_, respectively. Meanwhile, more porous carbon shells can be produced in PtFe 700_H_2_ than those in PtFe 900_H_2_ due to the effect of the annealing temperature. 

**Figure 2:**
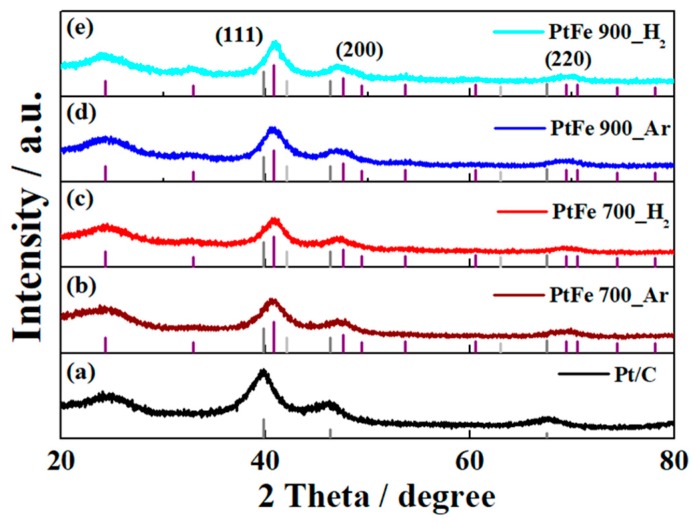
X-ray diffractometer (XRD)patterns of (**a**) Pt/C, (**b**) PtFe 700_Ar, (**c**) PtFe 700_H_2_, (**d**) PtFe 900_Ar, and (**e**) PtFe 900_H_2_. In the figures, the standard XRD peak patterns of fcc Pt (JCPDS# 87-0646, dark grey), ordered tetragonal PtFe (JCPDS# 65-1051, Ivory), and bcc Fe (JCPDS# 89-4186, light grey) are indicated.

To investigate the crystal structure of heat-treated PtFe catalysts, their XRD patterns were recorded and compared with commercial Pt/C as shown in Figure 2. The commercial Pt/C catalyst showed Bragg angles (2*θ*) at 39.8, 46.3, and 67.5° corresponding to (111), (200), and (220) planes (JCPDS# 87-0646) of the fcc structure of Pt (Figure 2a) [40]. On the other hand, in case of PtFe catalysts, the XRD peak positions were slightly shifted to 40.7, 47.4, and 69.7° compared to the fcc structure of Pt, in accordance with previous literature reports (JCPDS# 65–1051). The peak shift to higher angles in the XRD is attributed to the compressive strain of the Pt lattice by alloying with Fe. Since the heat-treated PtFe catalysts showed almost similar peak positions, it was revealed that they had a similar degree of alloying between Pt and Fe atoms. Furthermore, it was identified that the PtFe alloy catalysts have similar atomic ratios of Pt to Ru (Pt:Ru = 1:1) because their XRD peaks are positioned in the middle of those of Pt (JCPDS# 87-0646) and Fe (JCPDS# 89-4186). Notably, there was no significant change in the broadness or sharpness of the XRD peaks in all PtFe samples despite increasing the annealing temperature to 900 °C in Ar and H_2_ atmosphere, suggesting that the carbon shells formed during the annealing process definitely prevented severe agglomeration of PtFe nanoparticles on carbon supports. Meanwhile, as shown in Figure S4, N moieties in oleylamine used as surfactant in the synthesis of the PtFe_ASP sample were completely removed after the annealing process for heat-treated PtFe catalysts, obviously suggesting that there is no evidence of the formation of N-doped carbon or Fe−N as possible active sites for the ORR.

**Figure 3:**
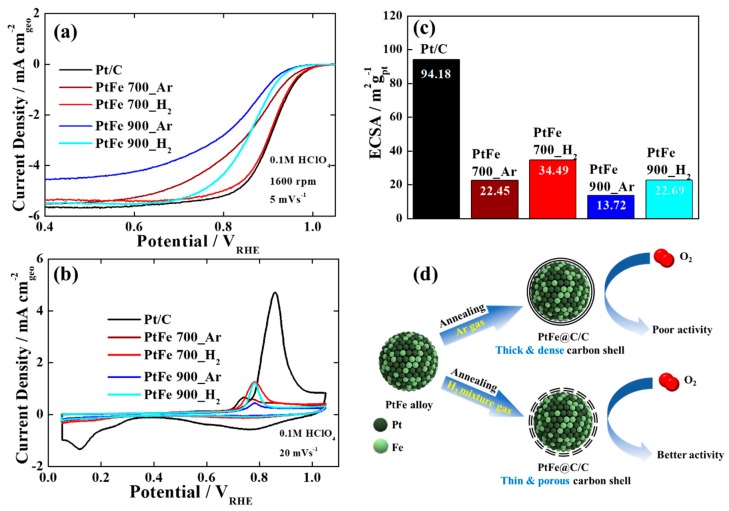
(**a**) oxygen reduction reaction (ORR) polarization curves, (**b**) CO stripping curves, and (**c**) electrochemcial surface area (ECSA) values of commercial Pt/C and heat-treated PtFe catalysts. (**d**) Illustration of the formation mechanism of carbon shell-coated PtFe catalysts and the difference in their ORR activities depending on the carbon shell structure.

To study the effects of the carbon shell formation on the electrocatalytic properties of PtFe nanoparticles, the ORR performances of the heat-treated PtFe catalysts were measured using a rotating disk electrode (Figure 3a). For comparison, a commercial Pt/C catalyst was also tested as a control. Interestingly, the heat-treated PtFe catalysts exhibited quite different ORR activities depending on the change in the carbon shell structures. First of all, PtFe 700_H_2_ showed the highest ORR performance comparable to that of commercial Pt/C in terms of a half-wave potential, E_1/2_, which is a crucial parameter to evaluate the electrocatalytic activity. The E_1/2_ for PtFe 700_H_2_ (0.91 V) was more positively shifted by ~50, ~70, and ~90 mV in comparison with PtFe 900_H_2_ (0.86 V), PtFe 700_Ar (0.84 V), and PtFe 900_Ar (0.82 V), respectively. Consequently, in the case of the carbon shell-coated PtFe catalysts annealed at Ar atmosphere, their ORR activities were significantly reduced compared to those heated at H_2_-mixed gas. In addition, with increasing the annealing temperature, the E_1/2_ was negatively shifted. 

To further understand the change in the catalytic activity of the heat-treated PtFe catalysts, CO stripping measurements were performed (Figure 3b). As expected, commercial Pt/C showed a prominent CO oxidation peak between 0.8 and 0.9 V due to highly exposed Pt surfaces. On the other hand, the heat-treated PtFe catalysts showed the negatively shifted CO oxidation peaks, indicating that alloying Pt with Fe can facilitate the CO oxidation reaction due to the bi-functional and ligand effects [41,42]. However, their CO oxidation peaks were greatly suppressed compared to the peaks of commercial Pt/C, indicating smaller or partially exposed active surfaces as a result of extensive carbon shell coverage. As shown in Figure 3c, PtFe 700_H_2_ showed the highest ECSA value among the carbon shell-encapsulated PtFe catalysts. Obviously, the result of the CO stripping test strongly suggests that more porous and thinner carbon shell structures can be produced when the PtFe nanoparticles are heated at relatively low temperature under H_2_-mixed gas atmosphere. The change in the carbon shell structure according to the annealing conditions can be understood to be for the following reasons. During the course of carbon shell formation, the strong interaction between H_2_ molecules and carbon disrupts the C−C bond formation on the metal nanoparticles and carbon layers is etched under H_2_ atmosphere, creating more defects and large pores [38,39]. In contrast, inert Ar gas does not affect the defect formation and carbon etching during the heat treatment, meaning dense and thick carbon shells can be formed [31,32,33,34,35,36,37]. As a result, in the catalysts with different carbon shells controlled by the annealing conditions, the trend of their ORR performance reveals that more porous and thinner carbon shells can provide easier access of O_2_ to the catalytically active sites for the ORR (Figure 3d).

**Figure 4:**
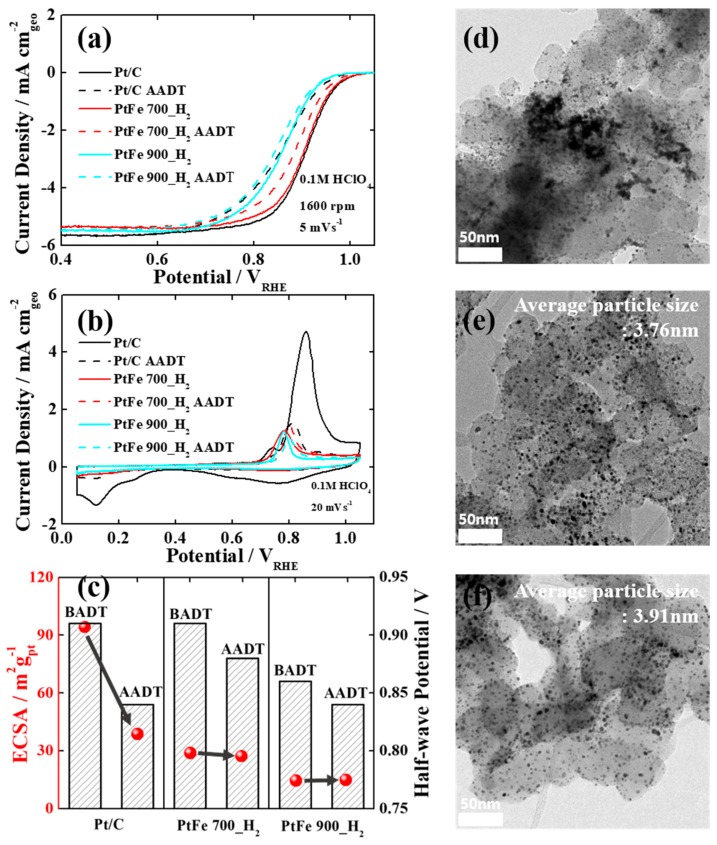
(**a**) ORR polarization and (**b**) CO stripping curves of commercial Pt/C, PtFe 700_H_2_, and PtFe 900_H_2_ before and after the ADTs. (**c**) Change in the ECSAs and E_1/2_ values of the tested catalysts, (**d**) TEM images of commercial Pt/C, (**e**) PtFe 700_H_2_, and (**f**) PtFe 900_H_2_ after the ADTs.

Lastly, to investigate the durability of the carbon shell-coated PtFe catalysts, we performed accelerated durability tests (ADTs) involving 5000 potential cycles between 0.6 and 1.1 V_RHE_. The ORR polarization and CO stripping curves of the catalysts were compared before and after the ADTs (Figure 4a,b). The comparison in the polarization curves clearly demonstrates that commercial Pt/C showed poor stability after the ADT with a sharp decline in E_1/2_ value by ~70 mV (Figure 4c). Furthermore, as shown in Figure 4b,c the tremendous reduction in the CO oxidation peak of commercial Pt/C after the ADT suggests a significant decrease in the number of active sites (from 94.18 to 38 m^2^/g), which clearly shows the poor stability of Pt. In addition, its corresponding TEM image after the ADT (Figure 4d) reveals that Pt nanoparticles were severely agglomerated on carbon supports. 

On the other hand, as shown in Figure 4a,c PtFe 700_H_2_ and PtFe 900_H_2_ exhibited superior long-term stabilities since the E_1/2_ values were changed a little even after the ADTs (ΔE_1/2_: ~30 and ~20 mV, respectively). The slightly reduced ORR activities might be attributed to the reduction of the alloying effects by the inevitable dissolution of some Fe atoms from the surface. As expected, their CO stripping curves and ECSAs were hardly changed (Figure 4b,c), which means that the active sites of the carbon shell-coated PtFe nanoparticles have not diminished due to the protective carbon shells, effectively preventing the dissolution and migration of metal nanoparticles [28]. In particular, for PtFe 900_H_2_ annealed at extremely high temperature, no change in the ECSA value before and after the ADT strongly supports the fact that the enhanced crystallization of the carbon shells ensures greater stability of the catalyst. The change in the calculated ECSA values for the catalysts before and after 5000 potential cycles is presented in Table S1. In addition, their corresponding TEM images (Figure 4e,f) recorded after the ADTs shows that there is no agglomeration or particle detachment. Furthermore, EDX analyses on PtFe 700_H_2_ and PtFe 900_H_2_ catalysts before and after the ADTs clearly demonstrates negligible dissolution of Fe atoms, maintaining the initial Pt:Fe (1:1) atomic ratios (Figures S5 and S6). Based on these observations, it is evident that the carbon shell-coated PtFe catalysts (PtFe 700_H_2_ and PtFe 900_H_2_) exhibit superior long-term stability in harsh ADT conditions compared to commercial Pt/C, mainly attributed to the formation of thin and porous carbon shell layers. Therefore, it can be concluded that the developed carbon shell encapsulation strategy provides not only excellent durability, but also spectacular ORR activity to Pt-based alloy nanoparticles.

## 4. Conclusions

We demonstrated an effective and new strategy to synthesize highly efficient and durable carbon shell-coated PtFe electrocatalysts by using a simple thermal decomposition and annealing process without additional polymer coating for the carbon shell formation. The carbon sources were effectively provided from acetylacetonates in metal precursors, and the carbon shell structures were rationally controlled by simply changing the annealing conditions (temperature and gas atmosphere). As a result, PtFe catalysts annealed at 700 °C under H_2_-mixed N_2_ gas showed much higher ORR activity and durability compared to commercial Pt/C catalyst due to the thin and porous carbon shell formation on PtFe nanoparticles. Furthermore, after increasing the annealing temperature, the carbon shell coated on the PtFe surface could improve the durability of the catalysts due to the enhanced crystallinity of the carbon shells. In conclusion, the hybrid catalyst structure with thin and porous carbon shells not only ensures easy access of the reactant molecules to the active sites for the ORR, but also prevents coalescence and detachment of metal nanoparticles from carbon supports under harsh PEMFC operating conditions. Therefore, we believe that the developed synthetic approach and catalyst structure will provide more insights into the development of highly active and durable fuel cell electrocatalysts for practical applications.

## Supplementary Materials

The following are available online at https://www.mdpi.com/2079-4991/9/10/1491/s1, Figure S1: TGA data to estimate the metal loading of (a) PtFe_ASP, (b) PtFe 700_Ar, (c) PtFe 700_H_2_, (d) PtFe 900_Ar, and (e) PtFe 900_ H_2_ catalysts; Figure S2: Low-resolution TEM images of (a) PtFe 700_Ar, (b) PtFe 700_ H_2_, (c) PtFe 900_Ar and (d) PtFe 900_ H_2_. Magnified TEM images of (e) PtFe 700_Ar, (f) PtFe 700_ H_2_, (g) PtFe 900_Ar and (h) PtFe 900_ H_2_; Figure S3: TEM images and particle size distribution of (a) commercial Pt/C, (b) PtFe_ASP, (c) PtFe 700_Ar, (d) PtFe 700_H_2_, (e) PtFe 900_Ar, and (f) PtFe 900_H_2_; Figure S4: N 1s core level XPS spectra of (a) PtFe_ASP, (b) PtFe 700 Ar, (c) PtFe 700 H_2_, (d) PtFe 900 Ar, and (e) PtFe 900 H_2_.; Table S1: Comparison of ECSA values of commercial Pt/C and different heat-treated PtFe samples (before and after 5000 potential cycles); Figure S5: TEM image and elemental maps of Pt, Fe, and C for (a) PtFe_ASP, (b) PtFe 700_H_2_, and (c) PtFe 900_H_2_. The corresponding EDX spectra of (d) PtFe_ASP, (e) PtFe 700_H_2_, and (f) PtFe 900_H_2_, repectively; Figure S6: TEM image and elemental maps of Pt, Fe, and C for (a) PtFe 700_H_2_ and (b) PtFe 900_H_2_ after the ADTs. The corresponding EDX spectra of (c) PtFe 700_H_2_ and (d) PtFe 900_H_2_, respectively.
