# Supplementary material for: Modulating Catalytic Activity and Durability of PtFe Alloy Catalysts for Oxygen Reduction Reaction Through Controlled Carbon Shell Formation
Source: Nanomaterials (Basel). 2019 Oct 19;9(10):1491. doi: 10.3390/nano9101491 (PMC6835385; doi:10.3390/nano9101491)
Supplement: Supplementary file 1 [file nanomaterials-09-01491-s001.pdf]

Supplementary Materials

Modulating Catalytic Activity and Durability of PtFe Alloy Catalysts for Oxygen Reduction Reaction Through Controlled Carbon Shell Formation

Youngjin Kim [†], A. Anto Jeffery [†], Jiho Min and Namgee Jung [*]

Graduate School of Energy Science and Technology (GEST), Chungnam National University, 99 Daehak-ro, Yuseong-gu, Daejeon 34134, Korea; yoongjin123@naver.com (Y.K.); jeffeeanto@gmail.com (A.A.J.); mjh9780@naver.com (J.M.)

* Correspondence: njung@cnu.ac.kr

† These authors contributed equally to this work.

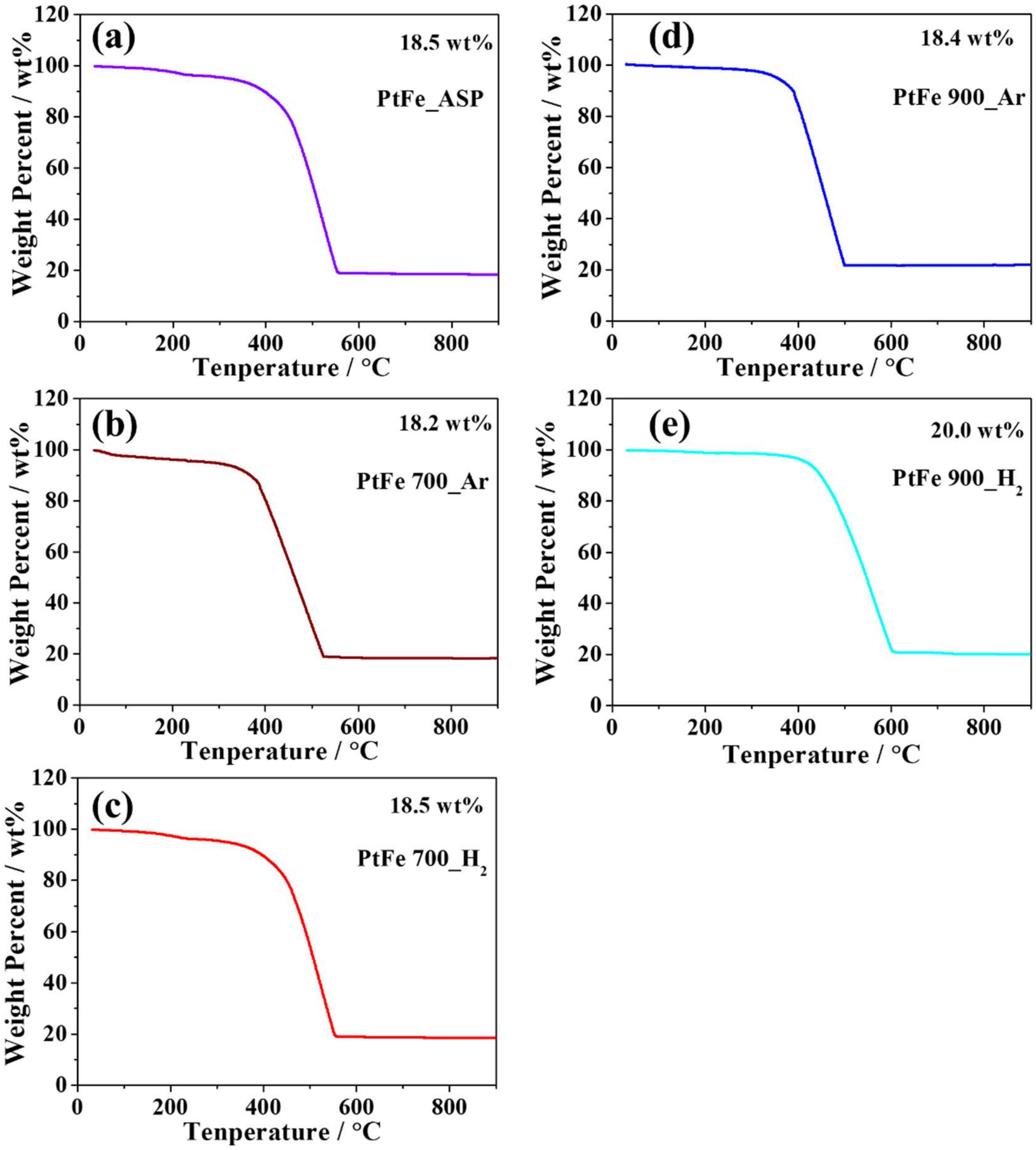

Figure S1. TGA data to estimate the metal loading of (**a**) PtFe_ASP, (**b**) PtFe 700_Ar, (**c**) PtFe 700_H_2, (**d**) PtFe 900_Ar, and (**e**) PtFe 900_H_2 catalysts.

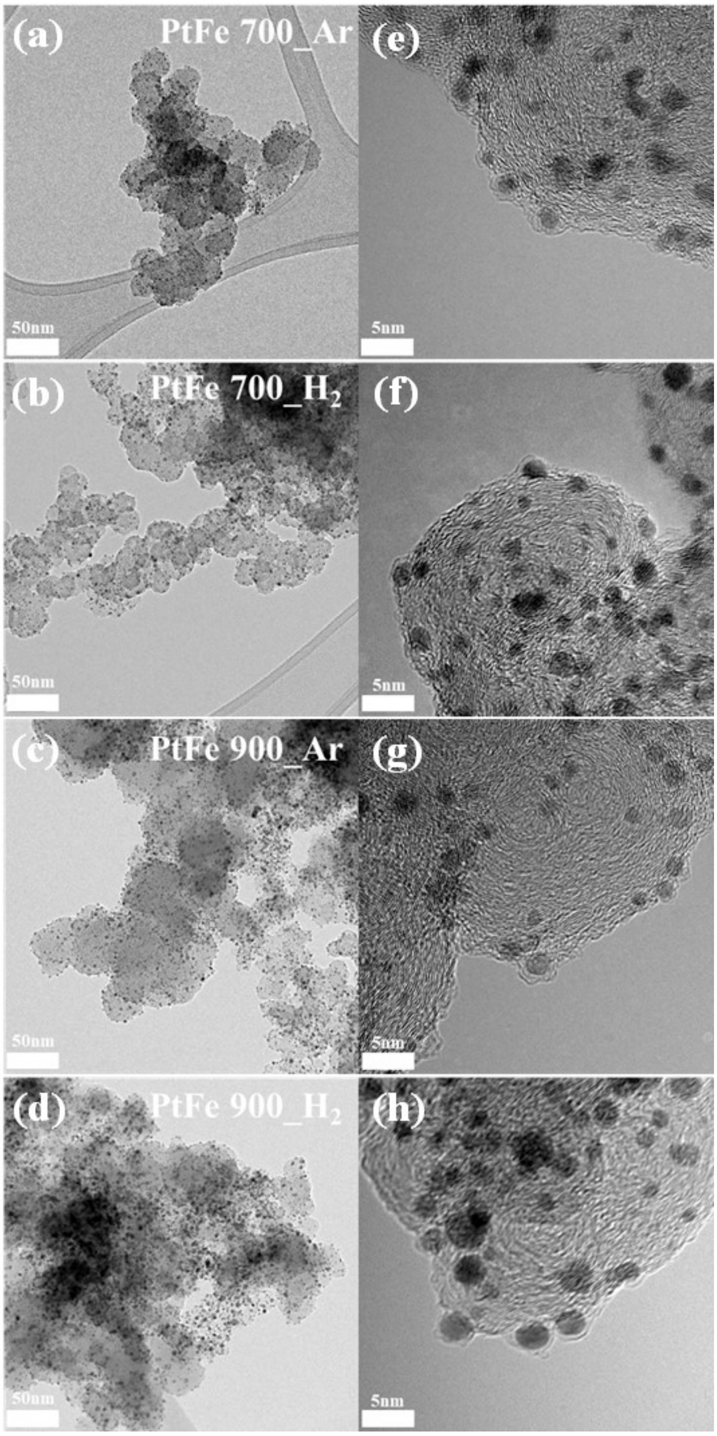

Figure S2. Low-resolution TEM images of (**a**) PtFe 700_Ar, (**b**) PtFe 700_H_2, (**c**) PtFe 900_Ar and (**d**) PtFe 900_H_2. Magnified TEM images of (**e**) PtFe 700_Ar, (**f**) PtFe 700_H_2, (**g**) PtFe 900_Ar and (**h**) PtFe 900_H_2.

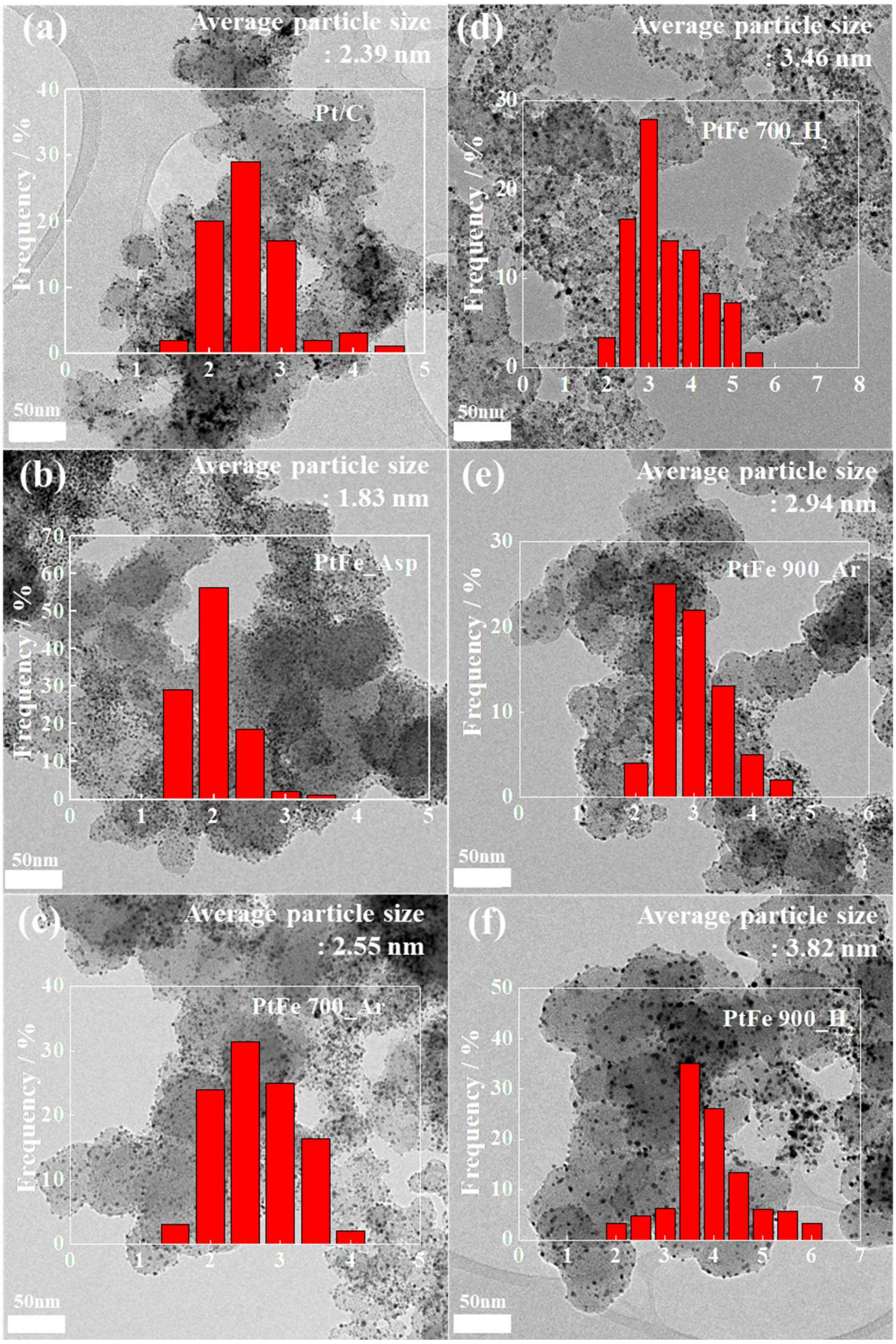

Figure S3. TEM images and particle size distribution of (**a**) commercial Pt/C, (**b**) PtFe_ASP, (**c**) PtFe 700_Ar, (**d**) PtFe 700_H_2, (**e**) PtFe 900_Ar, and (**f**) PtFe 900_H_2.

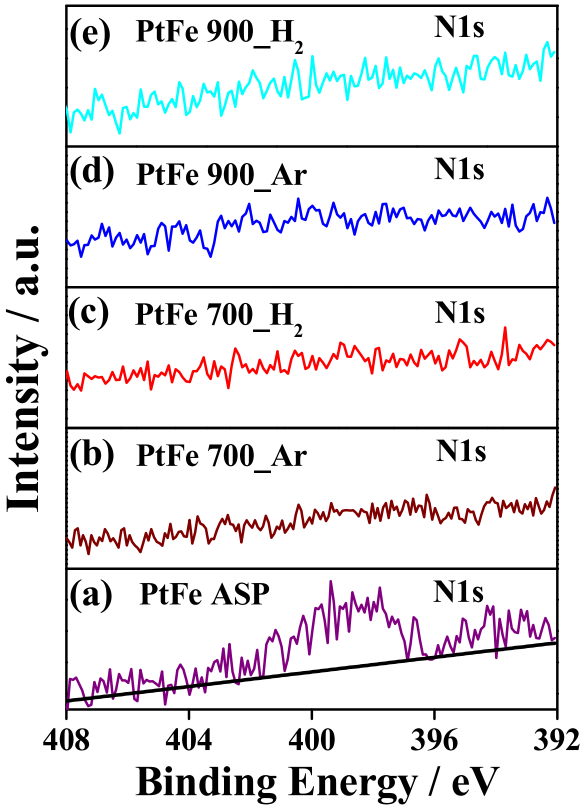

Figure S4. N 1s core level XPS spectra of (**a**) PtFe_ASP, (**b**) PtFe 700 Ar, (**c**) PtFe 700 H_2, (**d**) PtFe 900 Ar, and (**e**) PtFe 900 H_2.

Table S1. Comparison of ECSA values of commercial Pt/C and different heat-treated PtFe samples (before and after 5000 potential cycles).

	**Before ADT**	**After ADT**
**Pt/C**	94.18	38.63
**PtFe 700_Ar**	22.45	-
**PtFe 900_Ar**	13.72	-
**PtFe 700_H_2**	34.49	32.96
**PtFe 900_H_2**	22.69	23.05

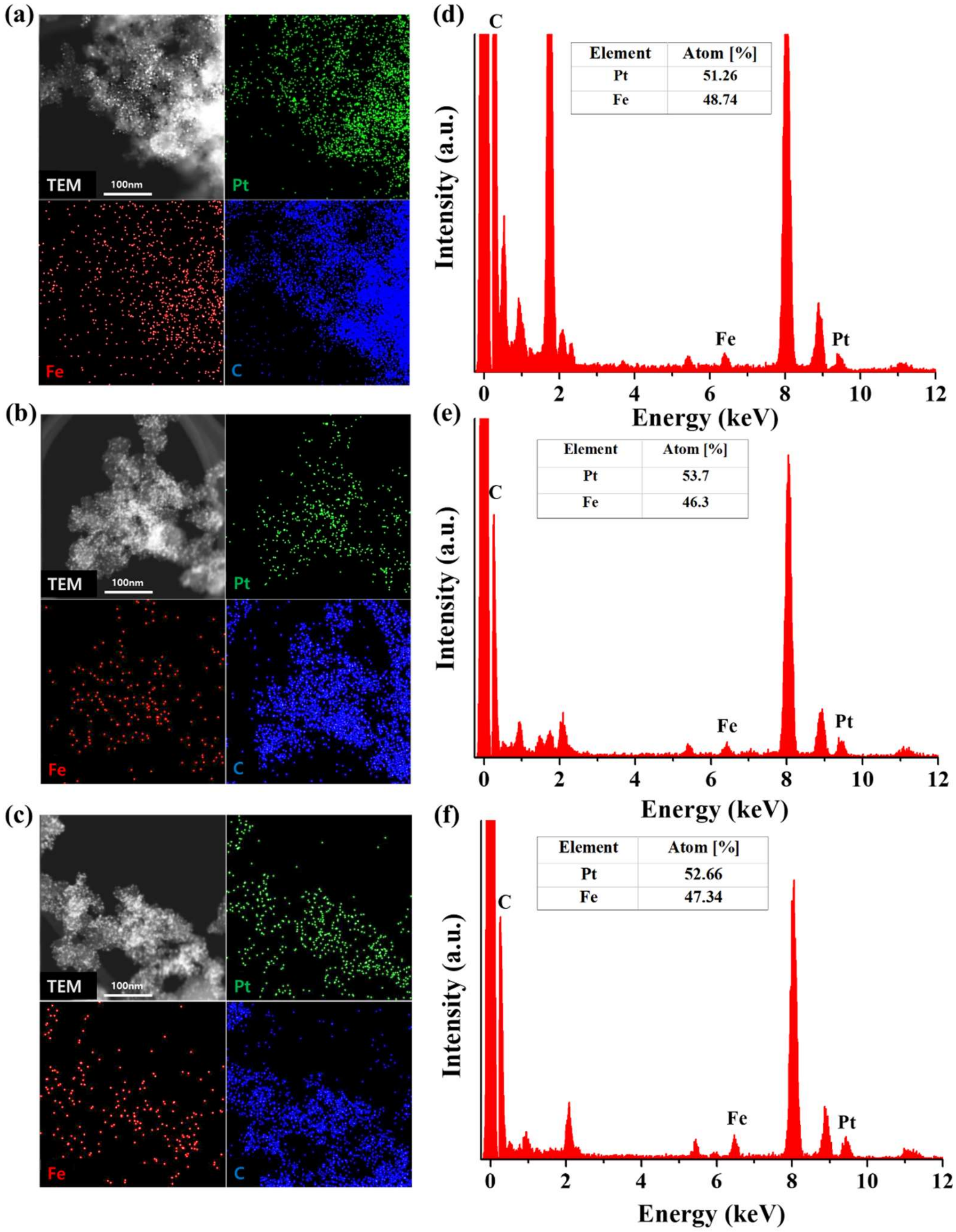

Figure S5. TEM image and elemental maps of Pt, Fe, and C for (**a**) PtFe_ASP, (**b**) PtFe 700_H_2, and (**c**) PtFe 900_H_2. The corresponding EDX spectra of (**d**) PtFe_ASP, (**e**) PtFe 700_H_2, and (**f**) PtFe 900_H_2, repectively.

Energy-dispersive X-ray spectrocopy (EDX) analysis were performed to identify elemental distribution of PtFe_ASP and different heat-treated PtFe catalysts. The elemental map of PtFe alloy catalyst revealed uniform distribution of elements, Pt and Fe. Furthermore, in the corresponding EDX spectra, it was confirmed that the prepared PtFe nanoparticles have near equivalent atomic ratios of Pt to Fe (Pt:Fe = 1:1).

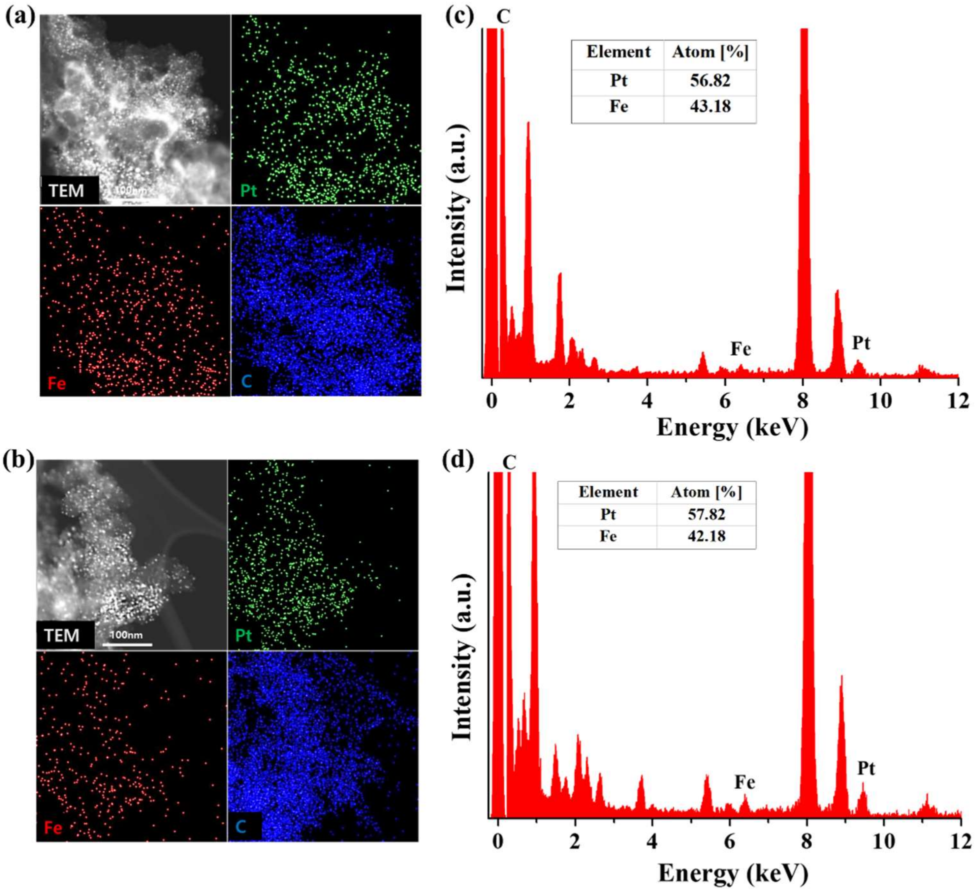

Figure S6. TEM image and elemental maps of Pt, Fe, and C for (**a**) PtFe 700_H_2 and (**b**) PtFe 900_H_2 after the ADTs. The corresponding EDX spectra of (**c**) PtFe 700_H_2 and (**d**) PtFe 900_H_2, respectively.

After the ADTs of PtFe 700_H_2 and PtFe 900_H_2 catalysts, their EDX mapping images showed no obvious change in the density of elemental distribution (Figure S6a and b). Notably, the corresponding EDX spectra (Figure S6c and d) revealed that the atomic ratios of Pt:Fe (1:1) were hardly changed even after the ADTs, indicating no significant loss of Pt or Fe atoms by dissolution.
